# Quercetin, a Lead Compound against Type 2 Diabetes Ameliorates Glucose Uptake via AMPK Pathway in Skeletal Muscle Cell Line

**DOI:** 10.3389/fphar.2017.00336

**Published:** 2017-06-08

**Authors:** R. Dhanya, A. D. Arya, P. Nisha, P. Jayamurthy

**Affiliations:** ^1^Agroprocessing and Technology Division, National Institute for Interdisciplinary Science and Technology, Council of Scientific and Industrial ResearchPappanamcode, India; ^2^Process Engineering and Environmental Technology, National Institute for Interdisciplinary Science and Technology, Council of Scientific and Industrial ResearchPappanamcode, India

**Keywords:** quercetin, L6 myotubes, AMPK, CaMMK, type 2 diabetes

## Abstract

Herein we investigated the molecular mechanism of action of the citrus flavonoid, quercetin in skeletal muscle cells (L6 myotubes). Taking advantage of protein kinase inhibitors, we proved that the effect of quercetin on 2-NBDG uptake in L6 myotubes was not through insulin signaling pathway, but through adenosine monophosphate kinase (AMPK) pathway and its downstream target p38 MAPK. An increase in the cellular AMP to ATP ratio on pretreatment may account for AMPK activation which was coupled with a transient change in mitochondrial membrane potential. In addition, quercetin triggered a rise in intracellular calcium suggesting that calcium-calmodulin mediated protein kinase (CaMKK) may also be involved. Quercetin shared a similar mechanism with the well-known drug metformin, highlighting it as a promising compound for the management of type 2 diabetes. The AMPK signaling pathway could contribute to correction of insulin resistance through bypassing the insulin-regulated system for GLUT4 translocation.

## Introduction

Diabetes mellitus is defined as a hyperglycemic condition arising due to insulin resistance or impaired insulin secretion. Controlling hyperglycemia in diabetes has been found to be difficult, as it requires high doses of oral antidiabetic agents and insulin. Alterations in signal molecules involved in insulin signaling (Kadowaki, 2000) evidently play a major role in insulin resistance seen in type 2 diabetes. Recent studies demonstrated that there exist a distinct intracellular signaling mechanism to stimulate glucose uptake and GLUT4 translocation. One stimulated by insulin through insulin receptor substrate-1/Phosphoinositol -3-kinase (IRS-1/P3K) (Lizcano and Alessi, 2002), the other by muscle contraction or exercise through the activation of AMPK, the exact mechanism of which is partially known (Sriwijitkamol et al., 2007). Both pathways increase the phosphorylation and activity of MAPK family components of which p38 MAPK participates in the full activation of GLUT4 (Cheng et al., 2006). P38 MAPK is said to be the downstream target of AMPK but it has also been suggested to have a role in the insulin stimulated glucose uptake (Xi et al., 2001; Somwar et al., 2002). Furthermore, overexpression of MKK3 and MKK6, the upstream regulatory kinases stimulated GLUT1 and GLUT4 transporters (Ryder et al., 2000). Alterations or defects in the insulin signal transduction pathway were found in diabetic patients associated with decreased levels of IRb, IRS-1, and PI3K (Kadowaki, 2000). Moreover, inhibition of PI3K, a key molecule involved in insulin signaling pathway, completely abolished insulin-stimulated uptake of glucose (Lai et al., 2012). Akt or Pkb is an important downstream target of insulin-stimulated glucose transport and metabolism. Also, insulin resistance associated with type 2 diabetes is mostly seen in skeletal muscle as it is the primary site of glucose as well as fatty acid utilization (DeFronzo and Tripathy, 2009). Therefore activation of AMPK in response to exercise has significant benefit for type 2 diabetics (Sriwijitkamol et al., 2007). For the same reason, therapeutic agents that overcome insulin resistance have received considerable attention. In recent years, several major insulin-sensitizing agents have been developed, including the thiazolidinediones (TZDs) (Sewter and Vidal-Puig, 2002) and metformin. Both the drugs exert their effects via activation of AMPK bypassing insulin signaling (Fryer et al., 2002). Understanding of the complete signal transduction involved in AMPK in skeletal muscle would offer significant pharmacologic benefits in the treatment of type 2 diabetes.

Last decade has witnessed enormous scientific studies on the heterogeneous class of molecules called phytochemicals. They are widely distributed in fruits, vegetables, beverages and in herbal remedies, but the use of natural products for the treatment of metabolic diseases has not been explored in detail (Marles and Farnsworth, 1995; Wang and Ng, 1999). Several traditional medicines have been reported for their antidiabetic effects (Mbikay, 2012), the molecular targets of such compounds remain uncharacterized. Flavonoids are an important component of most edible vegetables and fruits constituting a significant portion of the diet and have emerged as potential alternatives for treating diabetes, hyperlipidemia, and oxidative stress, involving multiple signaling pathways (Mbikay, 2012). In our previous study, we investigated the antidiabetic effect of quercetin in L6 myotubes under oxidative stress and concluded it to be a promising agent against Type 2 diabetes and its associated pathophysiology (Dhanya et al., 2014). In the present study, we attempt to reveal the molecular targets of quercetin in skeletal muscle cell line owing to its antidiabetic potential.

## Materials and Methods

Quercetin, Dulbecco’s modified Eagle’s media (DMEM), bovine serum albumin, streptomycin ampicillin–amphotericin B mix, rosiglitazone, insulin, quercetin, para nitrophenyl phosphate, 2-(7-Nitrobenz-2-oxa-1,3-diazol-4-yl) amino-2-deoxy-D-glucose (2-NBDG), ATP, ADP, AMP, HEPES, dithiothreitol, EDTA (Ethylene diamine tetra acetic acid), perchloric acid, dorsomorphin, wortmannin, primers, fura 2AM and JC-1 kit were purchased from Sigma–Aldrich Chemicals (St Louis, MO, United States); phospho-specific or pan-specific antibodies against AMPK, P38MAPK, GLUT 4, AKT, and IRS were purchased from Santa Cruz Biotechnology, United States. All other chemicals used were of standard analytical grade. L6 myoblast was obtained from National Centre for Cell Sciences, Pune, India.

### Cell Culture and Treatment

L6 myoblasts, Rat skeletal muscle cell lines were maintained in DMEM supplemented with10% FBS, 10% antibiotic-antimycotic mix at 37°C under 5% CO_2_ atmosphere. Cells were grown at a density of 1 × 10^5^cells/well. For differentiation, the cells were maintained in differentiation media containing 2% horse serum for 5–7 days. The concentration of quercetin was fixed at 10 and 100 μM and the period of incubation at 24 h based on our previous study (Dhanya et al., 2014).

### Inhibitor Studies

For the evaluation of the effects of kinase inhibitors, fully differentiated L6 myotubes were starved in serum free (SF) medium for 1.5 h, and then the inhibitors of AMPK (20 μM dorsomorphin) or PI3K (100 nM wortmannin) were added, and cells were incubated for 30 min. Cells were then treated with quercetin (10 and 100 μm) for 24 h in SF-medium with/without corresponding inhibitors. The glucose uptake assay was initiated by the addition of 2-NBDG uptake. The cells were then washed twice with cold phosphate-buffered saline (PBS) and the fluorescence in cells were acquired by confocal microscope (Pathway 855, BD Bioscience, San Jose, CA, United States) equipped with filters having excitation (490 nm) and emission (525 nm) in the FITC range. The changes in glucose uptake in cells were analyzed using BD Image Data Explorer software.

### Adenine Nucleotide Extraction and Measurement

Cells were washed with PBS and digested with trypsin. Total cell number were counted before cells are centrifuged at 8000 rpm for 3 min. Cell pellets were suspended in 3% perchloric acid and incubated on ice for 30 min. Within 1 h the pH of the lysate was adjusted between 6 and 8 with 2M KOH/0.3M MOPS. Precipitated salt was separated by centrifugation at 13000 × *g* for 10 min at 4°C. Aliquoted samples were stored at -80°C. Adenine nucleotide measurements were conducted by HPLC with a Phenomenex Gemini column (5mm, 0.46 cm × 15 cm, C18 110A) as previously described (Hahn-Windgassen et al., 2005). The nucleotides were detected spectrophotometrically at 259 nm and eluted at a flow rate of 1.0 ml/min. Internal standards (7.5 μM ATP, ADP, and AMP in ddH_2_O) were used to quantify the samples. The HPLC buffer contained 20 mM KH_2_PO_4_ and 3.5 mM K_2_HPO_4_ at pH 6.1.

### Assay for Mitochondrial Membrane Potential

Mitochondrial membrane potential was measured using mitochondrial staining kit, JC-1 following manufactures instructions. The kit uses the cationic, lipophilic dye, 5,5′,6,6′tetrachloro-1,1′,3,3′tetraethylbenzimidazolocarbocyanine iodide (JC-1). In normal cells, due to the electrochemical gradient, the dye concentrates in the mitochondrial matrix, where it forms red fluorescent aggregates (JC-1 aggregates). Change in mitochondrial membrane potential prevents the accumulation of the JC-1 and thus, the dye is dispersed throughout the entire cell leading to shift from red (JC-1 aggregates) to green fluorescence (JC-1 monomers). The cells after treatments were incubated with a JC-1 staining solution for 20 min at 37°C. The stain was washed off with PBS and examined under spinning disk microscope, and images were collected, and fluorescence intensity was also measured. For JC-1 monomers and aggregates the fluorescence were measured at 490/530 nm and 525/590 nm, respectively. Valinomycin (1 μg/mL) was used as positive control for the measurement of dissipation of mitochondrial membrane potential.

### Determination of Intracellular Calcium Levels

Differentiated L6 myoblast (5–7 days) cultured in 96 black well plates were treated with compounds of standardized concentrations for 24 h. Intracellular calcium levels were detected by staining the various groups with Fura-2AM for 20 min at 37°C. The stain was washed off with PBS and visualized under a spinning disk confocal microscope (Pathway 855, BD Bioscience, San Jose, CA, United States) at an excitation-emission wavelength of 350 and 510 nm, respectively.

### Quantitative Real Time PCR Analysis

Total RNA from pretreated L6 myotubes were isolated using trizol (Invitrogen Corp., Grand Island, NY, United States) according to the manufacturer’s protocol. One microgram RNA was reverse transcribed by Superscript VILO cDNA synthesis kit. The primer sequences for tested genes were; PPIA: Forward- 5′CAAAGTTCCAAAGACAGCAGAAA3′, Reverse- 5′CTGTGAAAGGAGGAACCCTTATAG3′, GLUT 4: Forward- 5′TCGTGTGGCAAGATGTGTAT3′, Reverse- 5′GTGCCTATGTATGTGGGAGAAA3′, Akt: Forward- 5′GAGCTGTGAACTCCTCATCAA3′, Reverse- 5′TCTCCATAGTCCTCTGGGTAAG3′, PI3K: Forward- 5′GTGGACAAAGCAGAAGCATTAC3′, Reverse- 5′ACCCTGTGTTCTTTGTCTAGTG3′; IRS: Forward- 5′GAGTTGAGTTGGGCAGAGTAG3′, Reverse- 5′CATGTAATCACCACGGCTATTTG3′, AMPK: Forward- 5′CCTATGAAGAGGGCCACAATAA3′, Reverse- 5′AGGTCACGGATGAGGTAAGA3′, CaMKK: Forward- 5′CGCTGGTTCCCACTCTTATC3′, Reverse- 5′GCTCCCTGACTCTTTGCTATT3′, MAPK: Forward- 5′CCCAAGGCCCAGAAATATGA3′, Reverse- 5′AAGAACTGGCTTGGAGATGG3′. Peptidylprolyl isomerase A (PPIA) was used as reference gene. Quantification was performed using a real-time PCR system (Bio-Rad, Hercules, CA, United States) with SYBR green. The cycling parameters were as follows: initial denaturation at 95°C for 1 min, followed by 40 cycles of denaturation at 95°C for 20 s, annealing at 60°C for 30 s, and extension at 72°C for 30 s. Results were presented as levels of expression relative to those of controls after normalization to PPIA using the 2^-ΔΔC_T_^ method. The analysis was carried out in triplicates.

### Western Blotting

Differentiated L6 myoblast (5–7 day) cultured in 6-well plates were treated with compounds of standardized concentrations for 24 h. L6 cells were homogenized in 1 ml of RIPA lysis buffer (25 mM Tris-HCl pH 7.4, 25 mM NaCl, 0.5 mM EDTA, 1% Triton-X-100, 0.1% SDS) for 30 min on ice and were centrifuged at 12000 rpm for 10 min. The supernatants were collected, and protease inhibitor cocktail was added (Roche, Mannheim, Germany). Supernatants were then stored at -80°C until analysis. Upon thawing, protein content was assayed by the bicinchoninic acid method standardized to bovine serum albumin (Roche, Laval, QC, Canada). Each sample were loaded at approximately 40 μg on 10% polyacrylamide mini gels and transferred to nitrocellulose membrane (Millipore, Bedford, MA, United States). Membranes were blocked for 1 h at room temperature and then incubated overnight at 4°C in blocking buffer with appropriate phospho-specific or pan-specific antibodies against AMPK, P38MAPK, GLUT4, AKT, IRS1, IRS2 (each at 1: 500 to 1:1000). Followed by incubation with anti-rabbit HRP-conjugated secondary antibodies at 1:1000 to 1:4000 (Santa Cruz, CA, United States). The revelation was performed using diaminobenzidine method (Sigma, United States). Gel band intensities were evaluated by densitometry analysis using Image densitometry software (Quantity one-4.6.7, 1D analysis software, Bio-Rad, United States).

### Immunofluorescence Assay

Following pretreatment with quercetin (10 and 100 μM) cells were washed with PBS and fixed for 5 min with 4% formaldehyde in PBS and quenched with 50 mM glycine in PBS for 10 min. Cells were blocked with 5% BSA in PBS for 1 h and incubated with monoclonal GLUT4 antibody solution (1:200 dilution in 1.5% BSA in PBS) at 4°C overnight followed by washing with PBS and 1 h incubation at room temperature with FITC-conjugated goat anti-mouse IgG secondary antibody (1:500 dilution, 1.5% BSA in PBS). Following PBS wash, images were acquired using laser scanning confocal microscope (Nikon A1R, Nikon Instruments, Melville, NY, United States) equipped with filters in the FITC range (i.e., excitation, 490 nm; and emission, 525 nm). Images were analyzed by NIS ELEMENTS software.

### Statistical Analysis

Results were expressed as means and standard deviations of the control and treated cells from triplicate measurements (*n* = 3) of three different experiments. Data were subjected to one-way ANOVA, and the significance of differences between means was calculated by Duncan’s multiple range test using SPSS, standard version 16 (SPSS, Inc.), and significance was accepted at *P* ≤ 0.05.

## Results

### Inhibitor Studies

**FIGURE 1:**
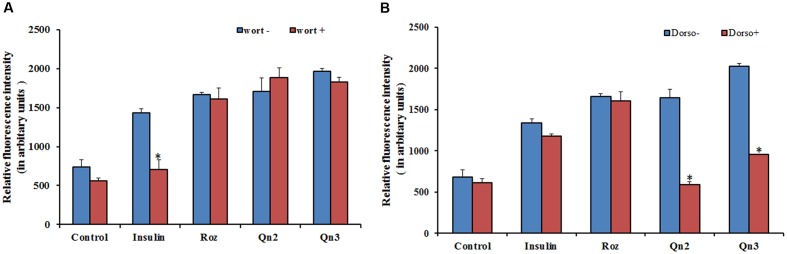
Effect of inhibitors on 2-NBDG uptake. **(A)** Effect of PI3K inhibitor, wortmannin, on 2-NBDG uptake in L6 myotubes. L6 myotubes pretreated with wortmannin followed by co-incubation with Rozi: rosiglitazone (100 nM); Insulin (100 nM); Qn (2, 3): quercetin (10 and 100 μM) for 24 h. Each value represents mean ± SD (standard deviation) from triplicate measurements (*n* = 3) of three different experiments. Significance test between different groups was determined by using one way ANOVA followed by Duncan’s multiple range test the significance accepted at *P* ≤ 0.05. ^∗^*P* ≤ 0.05 versus same groups with wortmannin. **(B)** Effect of AMPK inhibitor, Dorsomorphin, on 2-NBDG uptake in L6 myotubes. L6 myotubes pretreated with dorsomorphin followed by co-incubation with Rozi: rosiglitazone (100 nM); Qn (2, 3): quercetin (10 and 100 μM) for 24 h. Each value represents mean ± SD (standard deviation) from triplicate measurements (*n* = 3) of three different experiments. Significance test between different groups was determined by using one way ANOVA followed by Duncan’s multiple range test the significance accepted at *P* ≤ 0.05.^∗^*P* ≤ 0.05 versus same groups with dorsomorphin.

From our previous study, we got evidence for the possible antidiabetic potential of quercetin, as it stimulated 2-NBDG uptake in L6 myotubes via GLUT 4 translocation (Dhanya et al., 2014). As the effect of quercetin on glucose uptake was greater in magnitude than insulin, we assumed that it may employ other routes to attain its effect. To investigate whether the enhanced glucose uptake in L6 myotubes was mediated through PI3K activation, we examined the effects of selective inhibitors of PI3K and AMPK on 2-NBDG uptake. The effect of quercetin on 2-NBDG uptake was wortmannin insensitive as shown in **Figure 1A**, indicating that the insulin signaling pathway upstream of PI3K is not involved. Dorsomorphin treatment reduced the glucose uptake up to 80% as indicated in **Figure 1B** pointing to the conclusion that AMPK pathway has a significant role in 2-NBDG uptake induced by quercetin.

### Effect of Quercetin on AMP/ATP Ratio

**FIGURE 2:**
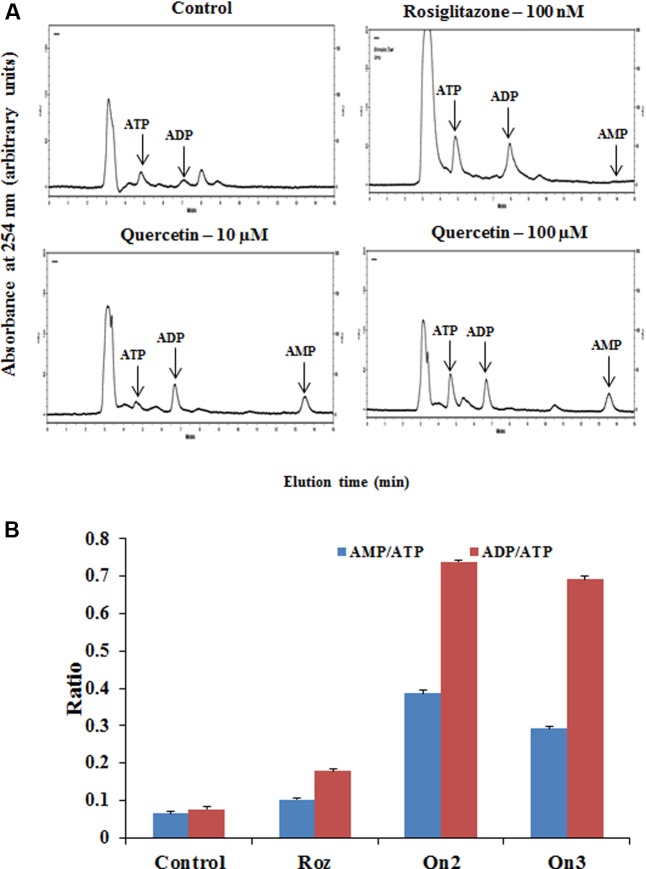
Analysis of adenine nucleotide levels in L6 myotubes. **(A)** Effect of quercetin on cellular nucleotide levels. The effect of different treatments (Rozi: rosiglitazone (100 nM); Qn (2, 3): quercetin (10 and 100 μM) on cellular nucleotides was determined by HPLC. In each case, a representative trace is shown. The position at which ATP, ADP and AMP standards eluted are approximately 5.4, 7.02, and 13.5 min, respectively, as indicated on each trace by arrows. **(B)** Adenine nucleotides ratio in L6 myotubes. The ratio of AMP to ATP and ADP to ATP are depicted in the above bar diagram. Adenine nucleotide concentrations and hence ratio changes on citrus flavonoids pretreatment. Rozi: rosiglitazone (100 nM); Qn (2, 3): quercetin (10 and 100 μM). Data are the mean results of three independent experiments ± SD.

The cellular AMP, ADP and ATP concentrations of L6 myotubes on pretreatment of quercetin (100 μM) was measured by HPLC as indicated in **Figure 2A**. Quercetin pretreatment caused twofold increase in both AMP to ATP as well as ADP to ATP ratio as indicated in **Figure 2B**. Interestingly, besides the AMP: ATP ratio, AMP, ADP and ATP concentrations significantly increased in treated myotubes. This may be the result of increased ATP utilization in these cells. The AMP: ATP ratio is an essential factor for cellular AMPK activation. As the cellular AMP: ATP ratio varies as the square of the ADP: ATP ratio (**Supplementary Table S1**), we used the ADP: ATP ratio as a surrogate measure.

### Transient Mitochondrial Depolarization

**FIGURE 3:**
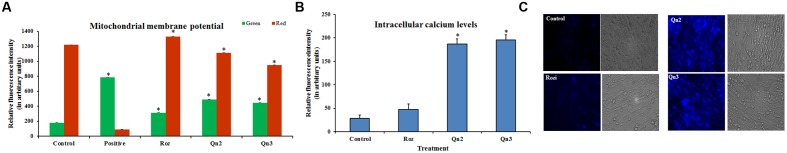
Change in membrane potential and intracellular calcium levels in L6 myotubes. **(A)** Fluorescence intensity analysis of red and green fluorescence. Relative fluorescence intensity was analyzed by BD Image Data Explorer software. There was a shift from red to green fluorescence on quercetin pretreatment indicating a transient change in mitochondrial transmembrane potential. Control: Untreated cells; Positive: Valinomycin treated, Rozi: rosiglitazone (100 nM); Qn (2, 3): quercetin (10 and 100 μM). Each value represents mean ± SD (standard deviation) from triplicate measurements (*n* = 3) of three different experiments. Significance test between different groups was determined by using one way ANOVA followed by Duncan’s multiple range test the significance accepted at *P* ≤ 0.05. ^∗^*P* ≤ 0.05 versus Untreated Control. **(B)** Fluorescence intensity analysis of intracellular calcium levels. Relative fluorescence intensity in L6 myotubes was determined by using Fura-2AM by BD Image Data Explorer software. Untreated cells (Control cells); Rozi: rosiglitazone (100 nM); Qn (2, 3): quercetin (10 and 100 μM). Significance test between different groups was determined by using one way ANOVA followed by Duncan’s multiple range test the significance accepted at *P* ≤ 0.05. ^∗^*P* ≤ 0.05 versus Untreated Control. **(C)** Fluorescent images of cytosolic calcium levels in L6 myotubes. Fluorescent images of cytosolic calcium levels in L6 myotubes were determined by using Fura-2AM. Untreated cells (Control cells); Rozi: rosiglitazone (100 nM); Qn (2, 3): quercetin (10 and 100 μM). Scale bar corresponds to 87 μM.

A transient depolarization was observed in L6 myotubes pretreated with quercetin (**Supplementary Figure S1**) and its relative fluorescence intensity was analyzed by BDI Explorer software as shown in **Figure 3A**. As we had earlier proved quercetin to be non-cytotoxic (≤10% toxicity at 100 μM for 24 h) (Dhanya et al., 2014), we ruled out the possibility of apoptosis or the opening of mitochondrial transition pore. Pretreatment of quercetin increased the AMP to ATP ratio which tightly correlated with its effect on mitochondrial membrane depolarization.

### Effect on Intracellular Calcium Levels

To assess the possible change in intracellular calcium levels in treated cells, a fluorescent indicator, Fura 2 AM was used. Our results showed that the flavonoid impaired calcium homeostasis by significantly increasing cytosolic calcium levels in L6 myotubes. Pretreatment of L6 myotubes with quercetin triggered an increase in cytosolic calcium levels as compared to control (**Figure 3C**), and its intensity was analyzed and represented in **Figure 3B**.

### Gene Expression Analysis

**FIGURE 4:**
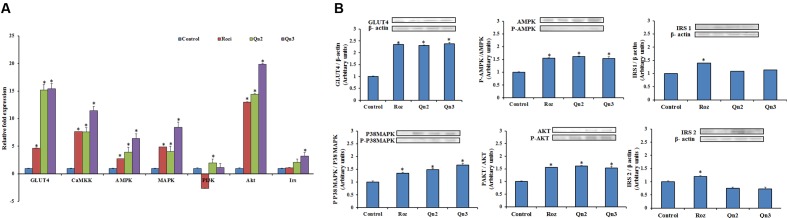
mRNA and protein expression in L6 myotubes on pretreatment of quercetin. **(A)** mRNA levels in L6 myotubes on pretreatment. The bar graph shows the mRNA levels (mean ± SE) of GLUT 4, CaMKK, AMPK, MAPK, PI3K, Akt and Irs in L6 myotubes on pretreatment of quercetin. The mRNA levels (arbitrary units) are expressed in relative to those of control cells. Quercetin (10 and 100 μM):Qn2 and Qn3. Significance test between different groups were determined by using one way ANOVA followed by Duncan’s multiple range test the significance accepted at *P* ≤ 0.05. ^∗^*P* ≤ 0.05 versus Untreated Control. **(B)** Protein expression in L6 myotubes. The effect of quercetin (10 and 100 μM): Qn2 and Qn3 on pretreatment for 24 h was comparable with that of the positive control rosiglitazone (100 nM). Significance test between different groups were determined by using one way ANOVA followed by Duncan’s multiple range test the significance accepted at *P* ≤ 0.05. ^∗^*P* ≤ 0.05 verses untreated control.

To evaluate the gene expression of the key signaling molecules involved in insulin and AMPK pathway, the mRNA levels were quantified by real-time PCR as shown in **Figure 4A**. Pretreatment of quercetin, at 100 μM showed a 14-fold increase in GLUT4 mRNA levels relative to control. There was a significant upregulation in Akt expression in L6 myotubes on quercetin pretreatment. CaMKK, AMPK, and MAPK, the key signaling molecules involved in AMPK were upregulated more than fivefold compared to control. Irs and PI3K showed onefold increase in mRNA levels on pretreatment compared to control.

### Western Blotting

To provide insight into the possible mechanism of glucose uptake stimulated by quercetin, the protein expression of IRS1, IRS2, AKT, P38MAPK and AMPK in L6 myotubes were investigated on pretreatment of quercetin by western blotting. Since the activation depends on the phosphorylation of the key signaling molecules, the expression of phosphorylated molecules were also examined. P38MAPK, AMPK, and AKT showed onefold increase in expression and phosphorylation of these proteins were also enhanced on pretreatment. GLUT4 expression increased to twofold in L6 myotubes, as expected there was a feeble expression of both IRS1 and IRS2 in L6 myotubes as shown in **Figure 4B**.

### Upregulation of GLUT4 in L6 Myotubes

GLUT4 upregulation in L6 myotubes was monitored by immunoassay with fluorescent labeled secondary antibody at 24 h pretreatment. Effect of quercetin (100 μM) on translocation was much higher than that of positive control as shown in supplementary data, suggested the end molecular mechanism behind the induction of AMPK pathway in L6 myotubes. The results obtained by quantifying immunologically labeled GLUT4 receptors at the surface of intact cells were depicted in **Supplementary Figure S2**.

## Discussion

The skeletal muscle has a paramount role in energy balance and is the primary tissue for insulin stimulated glucose uptake and disposal. Thus, it is considered to be an important therapeutic target tissue for non-insulin dependent diabetes mellitus (NIDDM) and cardiovascular disease. It account for approximately 80% of glucose absorption under insulin-stimulated conditions (Bailey and Turner, 2004) and a reduction in insulin-stimulated glucose uptake in skeletal muscles of type 2 diabetic patients has been observed both *in vitro* (Hakan et al., 2006) and *in vivo* (Rahman et al., 2007). Hence L6 myotubes have been widely used to investigate the mechanism of insulin and exercise stimulated glucose transport (Xiao et al., 2007). Using L6 myotubes, we had earlier reported the stimulatory effect of quercetin on glucose uptake (Dhanya et al., 2014). Herein we investigated the role of quercetin on AMPK signaling pathway for enhanced glucose uptake in L6 myotubes. AMPK is activated by various stimuli that cause an increase in AMP to ATP ratio. Binding of AMP or ADP to the AMPK- γ subunit causes a conformational change that promotes phosphorylation of Thr-172 by upstream kinases while inhibiting dephosphorylation by upstream phosphatases (Fryer et al., 2002; Hawley et al., 2002; Lin et al., 2010). Pretreatment of quercetin caused a twofold increase in the cellular AMP: ATP ratio which might have led to the activation of AMPK and attribute to the stimulation of glucose uptake in L6 myotubes. AMPK can also be activated by mechanisms independent of changes in AMP to ATP ratio (Hwang et al., 2009). AMPK can also be activated fivefold by the allosteric modification mediated by AMP and 50–100-fold by phosphorylation of α-subunit (Thr-172) by upstream kinases like tumor suppressor kinase (LKB1) and calcium-calmodulin mediated protein kinase (CaMKK). The effects of AMPK on the phosphorylation of acetyl-CoA carboxylase, are significantly inhibited by CaMKK inhibitor, STO-609, which confirms CaMKK as an important signaling molecule involved in AMPK pathway. Quercetin pretreatment triggered an increase in cytosolic calcium levels on L6 myotubes and upregulated the gene expression of CaMKK which suggested that CaMKK may also be involved in AMPK phosphorylation at Thr-172. Besides quercetin pretreatment resulted in a transient mitochondrial depolarisation in L6 myotubes that might have caused the inhibition of respiratory complex in L6 myotubes and therefore activated AMPK.

Quercetin pretreatment in L6 myotubes revealed a significant upregulation of mRNA levels of both AMPK and its downstream target P38 MAPK. AMPK has been earlier reported to play an important role in promoting p38 MAPK activation by inducing p38 MAPK autophosphorylation through interaction with the scaffold protein TAB1 in ischemic heart (Li et al., 2005). Though the role of P38 MAPK in glucose transport is contradictory (Sweeney et al., 1999; Fujishiro et al., 2001) there are reports on the impaired glucose uptake in skeletal muscles treated with P38 inhibitor (Somwar et al., 2000). Rosiglitazone downregulates PI3K as it belongs to thiazolidinedione class of drugs which are potentially useful in treating several cancers (Ramos-Nino and Littenberg, 2008) through both PPARγ-dependent and PPARγ-independent mechanisms. This concomitant upregulation of AMPK, P38 MAPK and AKT genes in L6 myotubes proved that the signaling pathway activated by quercetin partial overlaps with that of insulin signaling. GLUT4 translocation to the plasma membrane is a final common event as immunofluorescence analysis proved an increased expression of GLUT4 receptors on the plasma membrane of L6 myotubes (Dhanya et al., 2014). Herein we found that quercetin pretreatment caused a significant up regulation of both mRNA and protein levels of GLUT4 in L6 myotubes. The translocation of GLUT4 from an intracellular location to the plasma membrane through transverse tubules is thought to be the major mechanism by which exercise, AMPK activators, and insulin increase skeletal muscle glucose transport in mammals (Rahman et al., 2007).

Since both the signaling pathways were involved, we used specific protein kinase inhibitors (wortmannin and dorsomorphin) of PI3K and AMPK, to prove the exact molecular mechanism behind the up regulation of GLUT4 transporters and the subsequent increase in glucose uptake. The results from our study demonstrated that the effect of quercetin on L6 myotubes was not predominantly through PI3K signaling pathway but through AMPK pathway and its downstream target p38 MAPK. The mechanistic action of quercetin stimulating glucose uptake was found to be similar to that of resveratrol which promoted GLUT4 translocation through simultaneously enhancing phosphorylation of both AMPK and AKT (Miura et al., 2004; Breen et al., 2008; Deng et al., 2012) resulting in stimulation of glucose uptake in skeletal muscle cells. Quercetin induced glucose uptake in C2C12 cells was suggested to be mediated by increased AMPK signaling (Eid et al., 2010) though pAMPK was not directly measured. Similar effects have also been reported while using metformin (the well-known drug against T2DM) and various plant derived compounds. Metformin treatment provoked acute increases in the translocation of GLUT4 to the plasma membrane in mice gastrocnemius muscle and L6 myotubes (Sajan et al., 2010). Metformin has been known to alter mitochondrial ATP synthesis and change AMP to ATP ratio resulting in the activation of the enzyme. Many plants derived compounds have been reported to activate AMPK. These include resveratrol from red grapes, ginsenoside from Panax ginseng, curcumin from Curcuma longa, berberine from Coptis Chinensis, epigallocatechin gallate from green tea, the aflavin from black tea (Hoyer-Hansen and Jaattela, 2007), and hispidulin from snow lotus, another plant used in Chinese herbal medicine (Lin et al., 2010).

## Conclusion

**FIGURE 5:**
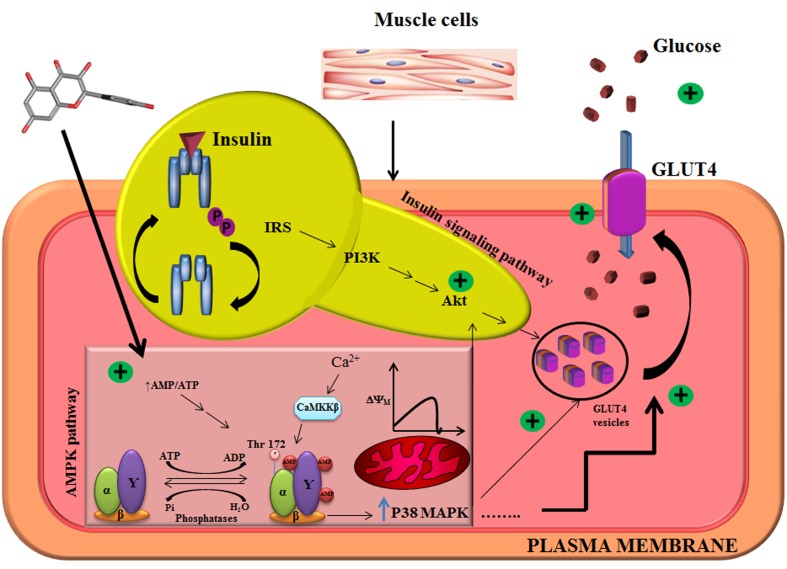
Illustration of mechanistic action of quercetin in L6 myotubes. Quercetin pretreatment in L6 myotubes enhanced AMP to ATP ratio which resulted in a transient change in mitochondrial membrane potential. There was also an associated increase in intracellular calcium level, which resulted in the activation of AMPK and its downstream target P38MAPK. Akt was also upregulated pointing to the conclusion that quercetin follows AMPK pathway, and it overlaps with insulin signaling pathway.

The present study report for the first time that quercetin imparts its antidiabetic potential via activating multiple therapeutic targets of type 2 diabetes, and it follows AMPK-P38 MAPK pathway to induce glucose uptake in L6 myotubes as illustrated in **Figure 5**. There may exist a cross talk between insulin signaling and AMPK pathway in upregulating GLUT4 expression as there was significant AKT mRNA expression in L6 myotubes on quercetin pretreatment. Our results also suggest the importance of modulating AMPK pathway for improved therapeutic approaches to type 2 diabetes and associated disorders.

## Author Contributions

RD designed, analyzed and performed the experiments and wrote the manuscript. AA performed the statistical analysis. PN and PJ designed and analyzed the experiments.

## Conflict of Interest Statement

The authors declare that the research was conducted in the absence of any commercial or financial relationships that could be construed as a potential conflict of interest. The reviewer YP declared a shared affiliation, though no other collaboration with the authors to the handling Editor, who ensured that the process nevertheless met the standards of a fair and objective review.

## Supplementary Material

**FIGURE S1** | Change in mitochondrial transmembrane potential in L6 myotubes on pretreatment of quercetin. Fluorescent images of cells stained with JC1 on pretreatment of untreated cells. Control: Untreated cells; Positive: Valinomycin treated; Rozi: rosiglitazone (100 nM); Qn (2, 3): quercetin (10 and 100 μM). Scale bar corresponds to 87 μM.

**FIGURE S2** | GLUT4 in L6 myotubes on pretreatment of quercetin. **(A)** Immunofluorescence images of GLUT4 in L6 myotubes. Upregulation of GLUT4 in differentiated L6 myoblast was visualized by Immunofluorescence assay. High resolution confocal images (40X) of Untreated L6 myotubes, Rosiglitazone (100 nM), and quercetin (100 μM, 24 h). Scale bar corresponds to 10 μM. **(B)** Fluorescence intensity analysis of GLUT4 in L6 myotubes on pretreatment of quercetin. The relative fluorescence intensity of GLUT4 was analyzed by NIS ELEMANTS software. Rozi: rosiglitazone (100 nM); Qn (2, 3): quercetin (10 and 100 μM) for 24 h. Significance test between different groups were determined by using one way ANOVA followed by Duncan’s multiple range test, ^∗^*P* ≤ 0.05.

**TABLE S1** | Adenine nucleotides from perchloric acid extracts of L6 myotubes were analyzed by HPLC: Adenine nucleotide concentrations and ratio changes on quercetin pretreatment. The areas under AMP, ADP, and ATP peaks were integrated to calculate AMP, ADP and ATP concentrations (pmol/105 cells). The values are the means ± SE of three independent measurements. ^∗^*P* < 0.05 verses control.
