# Supplementary figures and images for: Quercetin, a Lead Compound against Type 2 Diabetes Ameliorates Glucose Uptake via AMPK Pathway in Skeletal Muscle Cell Line
Source: Front Pharmacol. 2017 Jun 8;8:336. doi: 10.3389/fphar.2017.00336 (PMC5462925; doi:10.3389/fphar.2017.00336)

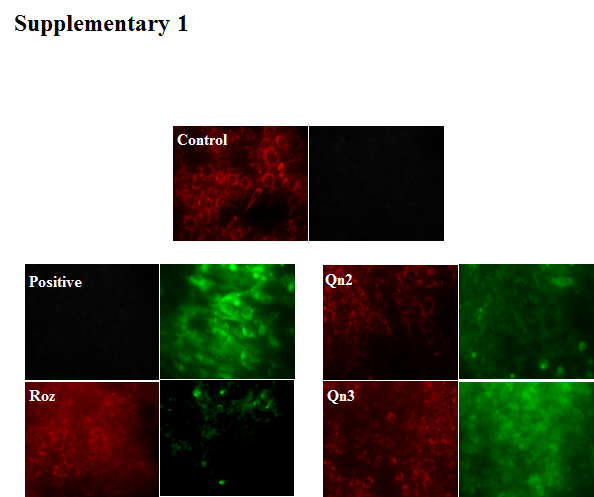

Supplement: Supplementary file 1 [file Image_1.TIF]

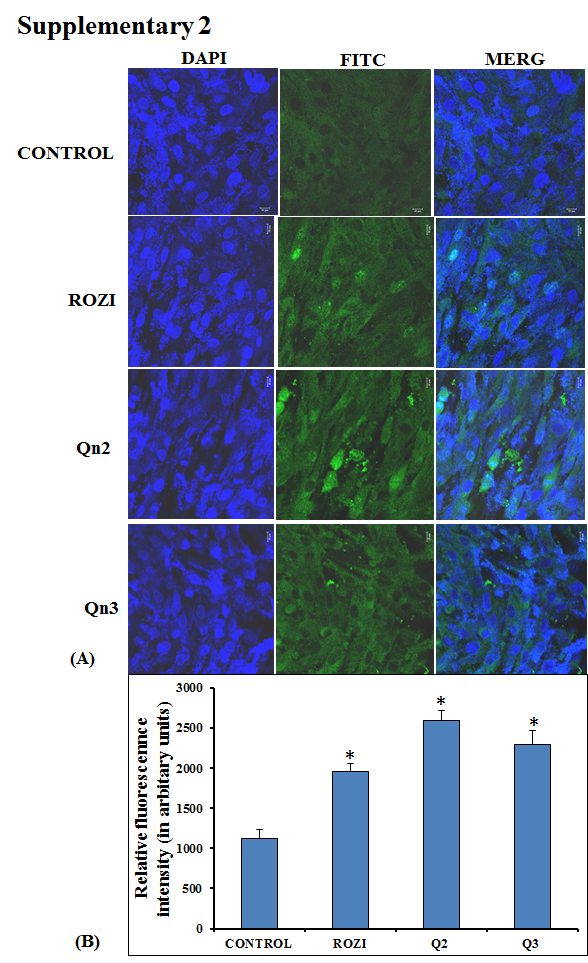

Supplement: Supplementary file 2 [file Image_2.tif]
