# Supplementary material for: Quercetin, a Lead Compound against Type 2 Diabetes Ameliorates Glucose Uptake via AMPK Pathway in Skeletal Muscle Cell Line
Source: Front Pharmacol. 2017 Jun 8;8:336. doi: 10.3389/fphar.2017.00336 (PMC5462925; doi:10.3389/fphar.2017.00336)
Supplement: Supplementary file 3 [file Table_1.DOC]

**Supplementary table 1: Adenine nucleotide concentrations on quercetin pretreatment analyzed by HPLC**

**Concentration (pmol /10^5^** cells)

| **Citrus flavonoids** | **AMP** | **ADP** | **ATP** |
| --- | --- | --- | --- |
| **Control** | 0.037937 | 1.783 | 0.590926 |
| **Rozi** | 0.601^*^ | 0.848 | 6.33^*^ |
| **Qn2** | 1.056* | 2.015* | 2.731* |
| **Qn3** | 0.684* | 1.17 | 2.984* |
|  |  |  |  |

Rozi: rosiglitazone (100 nM); Qn (2, 3): quercetin (10 & 100 μM). *P< 0.05 verses control***.***
